# Supplementary material for: P-TRAP: a Panicle Trait Phenotyping tool
Source: BMC Plant Biol. 2013 Aug 29;13:122. doi: 10.1186/1471-2229-13-122 (PMC3848748; doi:10.1186/1471-2229-13-122)
Supplement: Additional file 7 — P-TRAP source code. The Netbeans project that contains the source code of the application. [file 1471-2229-13-122-S7.zip › javadoc.zip!/javadoc/org-biolag-particleprocessor/org/biolag/particleprocessor/ParticlesAnalyizer.html]

ParticlesAnalyizer (ParticleProcessor)


JavaScript is disabled on your browser.


- Package
- Class
- Tree
- Deprecated
- Index
- Help

*org.biolag.particleprocessor 1.0*

- Prev Class
- Next Class

- Frames
- No Frames

- All Classes

- Summary:
- Nested |
- Field |
- Constr |
- Method

- Detail:
- Field |
- Constr |
- Method


org.biolag.particleprocessor

## Class ParticlesAnalyizer

- java.lang.Object
- - org.biolag.particleprocessor.ParticlesAnalyizer

- ---

    

  ```
  public class ParticlesAnalyizer
  extends java.lang.Object
  ```

  Given a set of particles detected on a binary image, this class is responsible for analyzing them.

  Author:
  :   faroq

  See Also:
  :   `Particle`,
      `ParticleFinder`

- - ### Nested Class Summary

    Nested Classes

    | Modifier and Type | Class and Description |
    | --- | --- |
    | `static class` | `ParticlesAnalyizer.CALC_METHOD` |
  - ### Constructor Summary

    Constructors

    Constructor and Description

    `ParticlesAnalyizer()`
  - ### Method Summary

    Methods

    | Modifier and Type | Method and Description |
    | --- | --- |
    | `static void` | `calcArea(java.util.ArrayList<Particle> particles)` |
    | `static void` | `calcAreaFromContour(java.util.ArrayList<Particle> particles)` |
    | `static void` | `calcBoundingBox(java.util.ArrayList<Particle> particles)` |
    | `static void` | `calcBounds(java.util.ArrayList<Particle> particles)` |
    | `static void` | `calcCircularity(java.util.ArrayList<Particle> particles)` |
    | `static void` | `calcCompactness(java.util.ArrayList<Particle> particles)` |
    | `static void` | `calcConcavePoints(java.util.ArrayList<Particle> particles, org.bioalg.imageprocessor.AImage img, int radius)` calculate the concave points using a 5x5 window centered on the contour point and it is neighbors and the outcome is the summation of the contour points in these three windows |
    | `static void` | `calcContour(java.util.ArrayList<Particle> particles, org.bioalg.imageprocessor.AImage img)` Find the contours of the particles using math morphology |
    | `static void` | `calcContour2(java.util.ArrayList<Particle> particles, org.bioalg.imageprocessor.AImage img)` a slow but more accurate method to get the contour of the particles |
    | `static void` | `calcCovixity(java.util.ArrayList<Particle> particles, org.bioalg.imageprocessor.AImage img, int radius)` Convexity measure based on Prof.Dr. |
    | `static void` | `calcCurves(java.util.ArrayList<Particle> particles, double minmumlength)` converts a given particle into two sets of concave and convex curves |
    | `static void` | `calcEllipticity(java.util.ArrayList<Particle> particles)` |
    | `static void` | `calcHeighAndWidth(java.util.ArrayList<Particle> particles)` |
    | `static void` | `calcNetArea(java.util.ArrayList<Particle> particles)` calculating the area using the green equation |
    | `static void` | `calcPosition(java.util.ArrayList<Particle> particles)` calc the center of mass of the particle |
    | `static void` | `calcPrimeter(java.util.ArrayList<Particle> particles)` |
    | `static void` | `calcPrimeter2(java.util.ArrayList<Particle> particles, org.bioalg.imageprocessor.AImage contourImage)` |
    | `static void` | `calcProp(java.util.ArrayList<Particle> particles)` |
    | `static void` | `calcProp(java.util.ArrayList<Particle> particles, double median, double mean, double mode)` Proportionality of the contour area to a given typical particle's area |
    | `static int` | `getbestkernelSize(org.bioalg.imageprocessor.AImage original, int mindisk, int maxdisk)` |
    | `static ConcavePoint` | `getConcavePoint(java.util.ArrayList<ConcavePoint> concavePoints, java.awt.Point point, Particle.CONTOUR_TYPE type)` |
    | `static java.util.ArrayList<ConcavePoint>` | `getConnectedCurve(ConcavePoint initial, java.util.ArrayList<ConcavePoint> allpoints, java.util.ArrayList<ConcavePoint> visited)` |
    | `static double` | `getMaxarea(java.util.ArrayList<Particle> particles)` |
    | `static double` | `getMeanarea(java.util.ArrayList<Particle> particles)` |
    | `static double` | `getMedianarea(java.util.ArrayList<Particle> particles)` |
    | `static double` | `getMinarea(java.util.ArrayList<Particle> particles)` |
    | `static double` | `getModearea(java.util.ArrayList<Particle> particles, double threshold)` |
    | `static double` | `getSTDarea(java.util.ArrayList<Particle> particles, double mean)` |
    | `static double` | `gettotalProp(java.util.ArrayList<Particle> particles)` |

    - ### Methods inherited from class java.lang.Object

      `clone, equals, finalize, getClass, hashCode, notify, notifyAll, toString, wait, wait, wait`

- - ### Constructor Detail


    - #### ParticlesAnalyizer

      ```
      public ParticlesAnalyizer()
      ```
  - ### Method Detail


    - #### getMedianarea

      ```
      public static double getMedianarea(java.util.ArrayList<Particle> particles)
      ```


    - #### getMeanarea

      ```
      public static double getMeanarea(java.util.ArrayList<Particle> particles)
      ```


    - #### getModearea

      ```
      public static double getModearea(java.util.ArrayList<Particle> particles,
                       double threshold)
      ```


    - #### getSTDarea

      ```
      public static double getSTDarea(java.util.ArrayList<Particle> particles,
                      double mean)
      ```


    - #### getMinarea

      ```
      public static double getMinarea(java.util.ArrayList<Particle> particles)
      ```


    - #### getMaxarea

      ```
      public static double getMaxarea(java.util.ArrayList<Particle> particles)
      ```


    - #### calcContour2

      ```
      public static void calcContour2(java.util.ArrayList<Particle> particles,
                      org.bioalg.imageprocessor.AImage img)
      ```

      a slow but more accurate method to get the contour of the particles

      Parameters:
      :   `particles` -
      :   `img` -


    - #### calcContour

      ```
      public static void calcContour(java.util.ArrayList<Particle> particles,
                     org.bioalg.imageprocessor.AImage img)
      ```

      Find the contours of the particles using math morphology

      Parameters:
      :   `particles` -
      :   `img` - a binary image to be used


    - #### calcPosition

      ```
      public static void calcPosition(java.util.ArrayList<Particle> particles)
      ```

      calc the center of mass of the particle

      Parameters:
      :   `particles` -


    - #### calcProp

      ```
      public static void calcProp(java.util.ArrayList<Particle> particles,
                  double median,
                  double mean,
                  double mode)
      ```

      Proportionality of the contour area to a given typical particle's area

      Parameters:
      :   `particles` -
      :   `median` -
      :   `mean` -
      :   `mode` -


    - #### calcProp

      ```
      public static void calcProp(java.util.ArrayList<Particle> particles)
      ```


    - #### gettotalProp

      ```
      public static double gettotalProp(java.util.ArrayList<Particle> particles)
      ```


    - #### calcCovixity

      ```
      public static void calcCovixity(java.util.ArrayList<Particle> particles,
                      org.bioalg.imageprocessor.AImage img,
                      int radius)
      ```

      Convexity measure based on Prof.Dr. Hans du Buf method

      Parameters:
      :   `particles` -
      :   `img` -
      :   `radius` - the radius where the crossproducts will be aggregated


    - #### calcCurves

      ```
      public static void calcCurves(java.util.ArrayList<Particle> particles,
                    double minmumlength)
      ```

      converts a given particle into two sets of concave and convex curves

      Parameters:
      :   `particles` -
      :   `minmumlength` -


    - #### getConcavePoint

      ```
      public static ConcavePoint getConcavePoint(java.util.ArrayList<ConcavePoint> concavePoints,
                                 java.awt.Point point,
                                 Particle.CONTOUR_TYPE type)
      ```


    - #### getConnectedCurve

      ```
      public static java.util.ArrayList<ConcavePoint> getConnectedCurve(ConcavePoint initial,
                                                        java.util.ArrayList<ConcavePoint> allpoints,
                                                        java.util.ArrayList<ConcavePoint> visited)
      ```


    - #### calcConcavePoints

      ```
      public static void calcConcavePoints(java.util.ArrayList<Particle> particles,
                           org.bioalg.imageprocessor.AImage img,
                           int radius)
      ```

      calculate the concave points using a 5x5 window centered on the contour point and it is neighbors
      and the outcome is the summation of the contour points in these three windows

      Parameters:
      :   `particles` -


    - #### calcCircularity

      ```
      public static void calcCircularity(java.util.ArrayList<Particle> particles)
      ```


    - #### calcEllipticity

      ```
      public static void calcEllipticity(java.util.ArrayList<Particle> particles)
      ```


    - #### calcCompactness

      ```
      public static void calcCompactness(java.util.ArrayList<Particle> particles)
      ```


    - #### calcArea

      ```
      public static void calcArea(java.util.ArrayList<Particle> particles)
      ```


    - #### calcNetArea

      ```
      public static void calcNetArea(java.util.ArrayList<Particle> particles)
      ```

      calculating the area using the green equation

      Parameters:
      :   `particles` -


    - #### calcAreaFromContour

      ```
      public static void calcAreaFromContour(java.util.ArrayList<Particle> particles)
      ```


    - #### calcPrimeter

      ```
      public static void calcPrimeter(java.util.ArrayList<Particle> particles)
      ```


    - #### calcPrimeter2

      ```
      public static void calcPrimeter2(java.util.ArrayList<Particle> particles,
                       org.bioalg.imageprocessor.AImage contourImage)
      ```


    - #### calcBoundingBox

      ```
      public static void calcBoundingBox(java.util.ArrayList<Particle> particles)
      ```


    - #### calcHeighAndWidth

      ```
      public static void calcHeighAndWidth(java.util.ArrayList<Particle> particles)
      ```


    - #### calcBounds

      ```
      public static void calcBounds(java.util.ArrayList<Particle> particles)
      ```


    - #### getbestkernelSize

      ```
      public static int getbestkernelSize(org.bioalg.imageprocessor.AImage original,
                          int mindisk,
                          int maxdisk)
      ```


- Package
- Class
- Tree
- Deprecated
- Index
- Help

*org.biolag.particleprocessor 1.0*

- Prev Class
- Next Class

- Frames
- No Frames

- All Classes

- Summary:
- Nested |
- Field |
- Constr |
- Method

- Detail:
- Field |
- Constr |
- Method
